# Implementation outcomes of video-observed oral fluid self-collection for drug testing in substance use disorder treatment: a pilot study

**DOI:** 10.3389/fpubh.2026.1833909

**Published:** 2026-05-19

**Authors:** Nicholas L. Bormann, Paul J. Jannetto, Tyler S. Oesterle

**Affiliations:** 1 Department of Psychiatry and Psychology, Mayo Clinic, Rochester, MN, United States; 2 Department of Laboratory Medicine and Pathology, Mayo Clinic, Rochester, MN, United States

**Keywords:** addiction, biospecimen, digital health, implementation science, oral fluid drug testing, remote monitoring, substance use disorder, telehealth

## Abstract

**Introduction:**

The integration of telehealth into clinical care has increased access to substance use disorder (SUD) treatment. However, drug screen monitoring has largely remained tied to in-person visits, preventing a fully virtual workflow. We evaluated the acceptability, appropriateness, feasibility, and usability of video-observed oral fluid (VOOF) self-collection as an approach to address this implementation gap.

**Methods:**

Participants receiving SUD treatment in outpatient (*n* = 13) or residential (*n* = 15) settings completed a video-recorded oral fluid self-collection protocol, followed by the Acceptability of Intervention Measure, Intervention Appropriateness Measure, Feasibility of Intervention Measure, and System Usability Scale.

**Results:**

Most participants had multiple SUDs (*n* = 23, 82.1%), and nearly all had co-occurring mental illness (*n* = 27, 96.4%). The majority completed study measures (*n* = 23, 82.1%) and returned collection kits (*n* = 25, 89.2%). Acceptability, appropriateness, and feasibility all exceeded established thresholds (>3), with respective means of 3.5 (SD 1.1), 3.8 (SD 1.1), and 3.4 (SD 1.3). Usability scores were below average and more variable (mean = 51.8, SD 20.4). Scores did not significantly differ by care setting.

**Discussion:**

This early-stage pilot found that VOOF self-collection was generally acceptable, appropriate, and feasible among individuals with SUD in real-world care settings. Usability scores were below average, with technical challenges related to video recording and upload likely contributing. Integrating VOOF within digital treatment platforms may streamline the user experience and improve usability while better aligning drug screen monitoring with the growth of telehealth in SUD treatment. This approach may benefit individuals experiencing transportation barriers and those in rural areas who live significant distances from testing sites.

## 1 Introduction

Monitoring for drug and alcohol use is a fundamental component of substance use disorder (SUD) treatment. The American Society of Addiction Medicine recommends utilizing drug testing as part of the initial comprehensive assessment and repeating it throughout the episode of care (1). Testing results can support patient-reported histories, while also providing additional information regarding potential safety risks and assessing if the current level of care remains appropriate (1).

Urine drug testing has historically been the most common approach and has occurred in person, either at the treatment site or by sending the individual to an off-site lab. With the expansion of telehealth services following the COVID-19 pandemic, many SUD treatment programs adjusted their delivery to provide more flexible care (2–6). This includes entirely virtual group-based treatment, hybrid groups where individuals join remotely alongside in-person participants, and the delivery of services through asynchronous digital interventions, such as mobile phone applications (i.e., apps) (5–12). Obtaining testing for these remote patients can be challenging, particularly in rural areas where healthcare shortages and large catchment areas exist (5, 13). Transportation issues, the lack of local testing sites, and the inability to easily share results between non-affiliated labs and treatment programs all contribute to friction in obtaining testing. Life circumstances, such as work schedules, childcare responsibilities, and weather conditions, can also impede patients from reaching on-site testing (2, 3, 14). This mismatch between telehealth-delivered treatment and in-person drug testing represents a practical implementation gap.

Remote toxicology monitoring has been explored as a means to help narrow this gap. For urine-based testing, collection protocols have included point-of-care testing where patients provide the sample during a video visit with study personnel, either live on-camera (15) or off-camera (5), as well as uploading a picture of the testing results for later review (16). Patients must have these kits available in advance, and if samples are not mailed for confirmatory analysis, results remain presumptive. Urine drug screening itself has been viewed as invasive and stigmatizing by some patients and providers (14, 17). Specific to telehealth and drug testing, patients’ perspectives have been mixed, including that the physical distance between patient and provider may increase or decrease stigma and trust depending on the overall clinical context (17, 18). Monitored urine collection can be particularly stigmatizing and embarrassing for patients (1, 19), and it is unclear if in-person or video monitoring differs in this regard.

Oral fluid drug testing is another objective means of assessment that has greater acceptance than monitored urine collection and does not require the same level of physical exposure (20, 21). Oral fluid testing is typically observed due to certification and procedural protocols, which help ensure the test is performed correctly and reduce the likelihood that samples may be tampered with (20, 21). By using a buffered solution and standard mailing packaging to maintain a stable environment, oral fluid samples can be collected at home and mailed to a lab for testing. Home-based biospecimen collection occurs elsewhere in healthcare, such as in chronic disease management and population screening (22–24). These assessments differ from drug testing, as the latter may carry implications for treatment duration, appropriateness for the level of care, and potential legal consequences. The incorporation of remote monitoring with home-based drug screening collection is therefore important to help ensure the individual providing the test is the intended individual; however, it must also be delivered in a patient-centered way that is mindful of stigma and privacy concerns.

Because implementation of remote toxicology monitoring involves both patient-facing and system-facing components, understanding patient perceptions is an essential early step before broader workflow integration. Past work has described challenges with digital augmentation of SUD care, including patient engagement and flow through care pathways (6, 25, 26), along with concerns regarding stigma, trust, and privacy with digital interventions (6, 17, 18, 27). However, patients routinely utilize digital interventions and biometric tracking for physical health, mental health, and SUD symptom monitoring and treatment, suggesting that a certain level of comfort exists (10, 28). Apps can leverage cameras on patients’ phones to provide a streamlined way to monitor oral fluid collection. However, just because it is technically feasible does not mean it would be acceptable to patients, particularly considering that drug testing has been experienced by some as punitive, intrusive, and stigmatizing.

Patients’ views on these matters are likely to influence intervention uptake and sustainability. To our knowledge, this is among the first studies to systematically assess patient-reported early implementation outcomes of video-observed oral fluid (VOOF) self-collection within a real-world SUD treatment-seeking population. While broader implementation includes full workflow assessment, from kit distribution and sample collection through laboratory processing and result reporting, this initial study focuses on patient-reported outcomes related to self-collection. This early-stage pilot was designed to assess the patient-facing aspects of this implementation, including outcomes of feasibility, acceptability, appropriateness, and usability.

## 2 Methods

### 2.1 Study design, sample, and recruitment

This was a prospective, early-stage feasibility and implementation pilot of VOOF self-collection and was not powered to assess efficacy or subgroup differences. Study recruitment occurred from 6/1/2025 to 2/20/2026. Inclusion criteria were adults aged 18 or older, with a SUD, able and willing to video-record themselves and upload the recording to the study team, fluent in English, and able to provide informed consent. Exclusion criteria included active, untreated mental health symptoms deemed by study investigators to impair the participant’s ability to safely engage in the study.

A convenience sample of participants was recruited through both outpatient and residential levels of care to facilitate enrollment. Patients in the outpatient setting were recruited from study investigators’ patient panels in southeast MN (NLB, TSO). Residential patients were recruited from the Mayo Clinic Intensive Addiction Program, a 30-day residential SUD treatment program in Rochester, MN. The *a priori* goal was to recruit an even balance between the two levels of care, with recruitment planned to continue until study funds were exhausted. Participant remuneration was $60 for study completion.

This study was reviewed and approved by the Mayo Clinic Institutional Review Board for human subjects research in accordance with institutional policies and federal regulations governing human participant research. The study was funded through internal departmental pilot funding.

### 2.2 VOOF self-collection procedure

**Figure 1:**
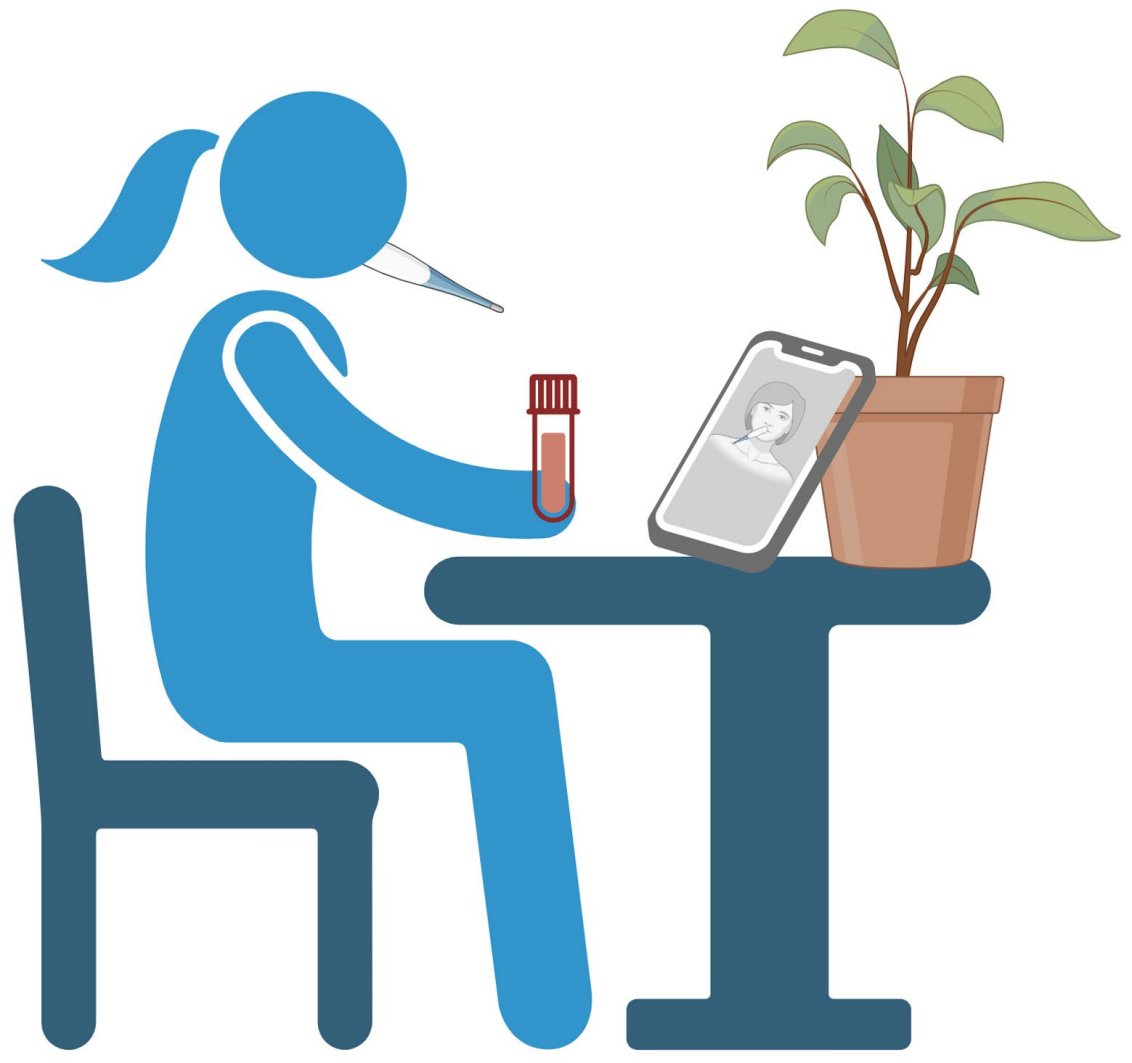
Participant instructions for video-observed oral fluid self-collection. Description: Visual aid provided to study participants demonstrating a potential setup for home-based video-observed oral fluid self-collection.

Participants were provided an oral fluid testing kit along with pre-paid packaging to return the completed kit. Written instructions asked participants to record themselves in a well-lit area where their hands and face were clearly visible. A link for uploading videos was included. Videos were securely transferred using industry-standard encryption to Mayo Clinic servers behind a Health Insurance Portability and Accountability Act-compliant firewall. For patients in residential settings, the main difference was that testing kits were provided directly and then returned to study staff after completion. Figure 1 was included in the instructions as a visual aid.

Information on how to perform the collection was also included, consistent with how the test is conducted in lab services. In brief, participants were instructed to discard any substance (i.e., food, gum, candy, etc.) from their mouths. If their mouth felt dry, they could rinse their mouth with up to 4 ounces of water. They were then asked to record themselves opening their mouths and sticking out their tongues to show that nothing was in their mouths. They then needed to wait 10 min without putting anything into their mouth, with continuous recording. Next, they placed the collection pad under their tongue, closing their lips around it, similar to a thermometer, tipping their head slightly downward to promote collection. Once the device indicator turned blue or the collection pad had been inserted for 10 min, the collection device was removed and placed into a transport tube that contained a buffer solution.

Additional information on the oral fluid panel, along with videos to prepare patients and lab staff for the collection process, can be found at: https://news.mayocliniclabs.com/therapeutics/oral-fluid.

### 2.3 Measures and analytical plan

The primary study focus was on standard implementation outcomes; a secondary focus was kit return rates. The usability of self-collection and recording was measured with the System Usability Scale (SUS) (29). The SUS assesses ten items using a 5-point Likert scale (Strongly Agree to Strongly Disagree), with a range of 0 to 100, and is widely used in implementation science. A SUS of 68 is considered average, with lower scores indicating below average usability (29).

The acceptability, appropriateness, and feasibility of self-collection were evaluated with the validated Acceptability of Intervention Measure (AIM), Intervention Appropriateness Measure (IAM), and Feasibility of Intervention Measure (FIM) (30, 31). Each scale consists of four items rated on a 5-point Likert scale (Strongly Agree to Strongly Disagree). Scores range from 1 to 5; an average score of 3 is considered passable.

Study scale composite scores were first assessed for normality within each recruitment site (residential versus outpatient) using Shapiro–Wilk tests and visual inspection. Composite scores were then compared by recruitment site using a two-sided Welch’s two-sample *t*-test. Given the small sample size, nonparametric Mann–Whitney *U* tests were conducted as sensitivity analyses. Differences in scale completion and kit return rates were evaluated using two-sided Fisher’s exact tests. No data imputation was performed. All analyses were conducted using R version 4.3.2 (32).

## 3 Results

Participant characteristics are shown in Table 1. A total of 28 participants (*n* = 13, female) consented to participate in the study. Mean age was 39.6 years (SD 10.1, range: 18–62). Recruitment was nearly even, with 13 participants from the outpatient setting and 15 participants from the residential setting. Most participants had at least two SUDs (*n* = 23, 82.1%). Alcohol use disorder was the most common SUD (*n* = 24, 85.7%), followed by tobacco (*n* = 17, 60.7%) and cannabis use disorder (*n* = 10, 35.7%). Nearly all participants (*n* = 27, 96.4%) had a co-occurring mental illness, with most having at least two (*n* = 19, 67.9%). Anxiety disorder was the most common (*n* = 18, 64.3%), followed by major depressive disorder (*n* = 16, 57.1%) and posttraumatic stress disorder (*n* = 9, 32.1%).

**Table 1:** Study participant demographics and characteristics.

Variable	Quantity
Female (%)	13 (46.4)
Age, mean (SD; years)	39.5 (10.1)
Outpatient (%)	13 (46.4)
Race	
White, *n* (%)	26 (92.3)
Black, *n* (%)	1 (3.6)
Declined to disclose, *n* (%)	1 (3.6)
Ethnicity	
Not Hispanic or Latino, *n* (%)	26 (92.3)
Hispanic or Latino, *n* (%)	1 (3.6)
Declined to disclose, *n* (%)	1 (3.6)
Education	
<12th grade, *n* (%)	1 (3.6)
12th grade or equivalent, *n* (%)	10 (35.7)
Some college, *n* (%)	5 (17.9)
College graduate or greater, *n* (%)	10 (35.7)
Any substance use disorder, *n* (%)	28 (100)
Alcohol use disorder, *n* (%)	24 (85.7)
Cannabis use disorder, *n* (%)	10 (35.7)
Cocaine use disorder, *n* (%)	4 (14.3)
Inhalant use disorder, *n* (%)	1 (3.6)
Methamphetamine use disorder, *n* (%)	6 (21.4)
Opioid use disorder, *n* (%)	4 (14.3)
Sedative use disorder, *n* (%)	1 (3.6)
Tobacco use disorder, *n* (%)	17 (60.7)
Any mental illness, *n* (%)	27 (96.4)
Attention-deficit/hyperactivity disorder, *n* (%)	2 (7.1)
Anxiety disorder, *n* (%)	18 (64.3)
Bipolar spectrum disorder, *n* (%)	3 (10.7)
Gambling disorder, *n* (%)	1 (3.6)
Major depressive disorder, *n* (%)	16 (57.1)
Personality disorder, *n* (%)	2 (7.1)
Posttraumatic stress disorder, *n* (%)	9 (32.1)

For study scales, 23 participants (82.1%) completed all four, while the remainder did not complete any; there were no statistically significant differences between outpatient (*n* = 9) and residential (*n* = 14) scale completion (*p* = 0.15). For kit return, 25 participants (89.2%) returned their collection kits; there were no statistically significant differences between outpatient (*n* = 13) and residential (*n* = 12) kit return (*p* = 0.23).

For implementation scales, mean AIM was 3.5 (SD 1.1; 95% CI: 3.0–3.9), mean IAM was 3.8 (SD 1.1; 95% CI: 3.3–4.2), and mean FIM was 3.4 (SD 1.3; 95% CI: 2.9–4.0). The mean SUS was 51.8 (SD 20.4; 95% CI: 43.0–60.7), indicating below-average usability. Item-level responses (Supplementary Table 1) showed that the lowest-rated items reflected perceptions of inconsistency, complexity, and cumbersome use, while the highest-rated items reflected strong perceived integration of functions and ease of learning.

Shapiro–Wilk tests did not indicate significant deviations from normality for any composite measure (*p*’s > 0.24). There were no statistically significant differences observed between recruitment groups across all four scales (see Table 2). Confidence intervals for mean differences were wide, and all included zero. Nonparametric Mann–Whitney *U* tests yielded consistent results (*p*’s > 0.29). Item-level responses for the AIM, IAM, and FIM can be seen in Table 3.

**Table 2:** Study scales compared by recruitment site.

Measure	Outpatient mean (SD)	Residential mean (SD)	Mean difference (95% CI)^a^	*p*-value^a^
Acceptability of intervention measure	3.2 (1.1)	3.6 (1.1)	−0.4 (−1.61 to 0.81)	0.48
Intervention appropriateness measure	3.3 (1.1)	4.1 (1.1)	−0.8 (−1.94 to 0.39)	0.17
Feasibility of intervention measure	3.2 (1.3)	3.6 (1.3)	−0.4 (−1.63 to 0.93)	0.56
System usability scale	48.6 (25.3)	53.9 (17.3)	−5.3 (−26.2 to 15.5)	0.59

a Welch’s two-sample *t*-test.

Description: Comparison of implementation outcome scale scores between outpatient and residential participants.

**Table 3:** Item-level responses for the acceptability, intervention appropriateness, and feasibility measures.

Scale	Question	Mean (SD)
Acceptability of intervention measure	Following the instructions and recording meets my approval	3.6 (1.3)
	Following the instructions and recording is appealing to me	3.5 (1.3)
	I like following the instructions and recording	3.2 (1.5)
	I welcome following the instructions and recording	3.5 (1.3)
	–	3.5 (1.1)^a^
Intervention appropriateness measure	Following the instructions and recording seems fitting	3.9 (1.1)
	Following the instructions and recording seems suitable	4.0 (1.2)
	Following the instructions and recording seems applicable	3.5 (1.4)
	Following the instructions and recording seems like a good match	3.7 (1.3)
	–	3.8 (1.1)^a^
Feasibility of intervention measure	Following the instructions and recording seems implementable	3.7 (1.4)
	Following the instructions and recording seems possible	3.3 (1.5)
	Following the instructions and recording seems doable	3.4 (1.6)
	Following the instructions and recording seems easy to use	3.3 (1.5)
	–	3.4 (1.3)^a^

a Scale mean.

Description: Responses were rated on a 5-point Likert scale ranging from strongly disagree to strongly agree. A score of 3 on each scale is considered passable.

## 4 Discussion

We found that VOOF self-collection was generally acceptable, appropriate, and feasible among individuals engaging in SUD treatment. In contrast, usability scores were below average, highly variable, and fell short of commonly accepted benchmarks. Despite this, participants were able to complete and return testing kits from real-world care settings. This pattern of adequate feasibility alongside below average usability may suggest that participants were willing to engage with the process even when the experience was not optimal. While improvements are needed before broader integration into routine SUD care, these findings support the approach’s acceptability and perceived feasibility from the patient perspective.

Telehealth delivery has become increasingly normalized within SUD treatment, with many components of care routinely completed remotely (2, 4). Within this context, it is unsurprising that the mean AIM, IAM, and FIM scores were above 3, suggesting that participants agreed the approach made sense within their treatment plan and was workable for them. Participants voiced frustration with some technical aspects, however. Positioning their phones for recording, ensuring adequate lighting, saving and uploading video files, and navigating inconsistent Wi-Fi or cellular connectivity were reported to study staff as implementation challenges. These friction points aligned with the SUS domains, particularly perceptions of complexity, inconsistency, and cumbersome use. While several of the voiced barriers were infrastructure-related and appeared to more commonly occur among participants in the residential unit, they likely contributed to lower usability and negatively impacted the participant experience.

Past research has evaluated remote drug screen self-collection in several ways. Participants have asynchronously provided point-of-care urine drug testing at home, uploading pictures of their results for staff to review (16). Participants have also provided urine drug screens during live video sessions, either off-camera (5) or on-camera via a profile view so staff could see the urine leaving the body and entering the collection cup (15). Despite being exposed, participants who provided urine on video rated the question “comfort level and your sense of respect” during the interaction (in one question) uniformly high (15). Alternatively, observed urine collection has also been experienced by patients as stigmatizing and an invasion of privacy, and is not broadly recommended in routine addiction care (1, 33). Additionally, opioid treatment programs in the United States that have ≥ 90% of clients participate in observed urine drug screens have a significant association with decreased overall retention in care compared to those that do not implement this monitoring (34). While some individuals may feel uncomfortable having their faces on camera, it is possible that they may prefer a monitored oral fluid collection over a monitored urinary collection. To our knowledge, this has not been evaluated previously.

While less is known about biospecimen collection, video monitoring has been previously evaluated for treatment adherence. In opioid use disorder, having patients video record themselves taking buprenorphine was found feasible and acceptable (*n* = 14; no validated implementation scales were used), with daily videos being submitted by participants 72% of the time (35). In a randomized clinical trial (*n* = 78), direct observation of buprenorphine through video monitoring did not improve treatment adherence or illicit substance use; the authors noted that drop-out may have contributed to negative findings (36). In a cohort of individuals with stimulant use disorder on extended-release injectable naltrexone (*n* = 49), 86.6% of doses of once-daily oral extended-release bupropion over 8 weeks were verified by participants uploading videos of them taking the medication through an app. Participants found the app easy to use, and that uploading videos helped improve their treatment adherence (37). In tuberculosis treatment, which is more common among individuals with a SUD than the general public, video observed treatment of antibiotics was found to require less time, less cost, and lead to superior adherence when compared to in-person observation of treatment (38). Collectively, these studies suggest that video-based verification is technically feasible across multiple clinical contexts, but the quality of the individual’s experience and engagement may depend on specific operational features (25). Integrating asynchronous video or photo upload within user-friendly digital platforms, as opposed to standalone workflows, appears to reduce friction.

These findings have particular relevance for geographically dispersed addiction treatment settings, where travel burden and workforce shortages limit the feasibility of frequent in-person monitoring (2, 4). App-based digital interventions have increasingly been explored as ways to extend the capacity of treatment teams beyond the traditional clinic (10, 11, 25, 39). Previously mentioned studies that incorporated remote monitoring directly within apps had greater levels of intervention engagement and reported acceptability by patients (16, 35, 38). Integrating these functions into a single digital platform may reduce technical barriers by simplifying file capture, storage, and transmission, while allowing treatment teams to review submissions live or asynchronously. This streamlining may also improve usability by reducing the number of steps and workflow complexity for the patient. In this context, incorporating VOOF self-collection within digital care platforms may help align drug screening monitoring workflows with the broader shift toward telehealth-delivered addiction treatment. The impact of this may be greatest in rural settings.

Several practical improvements emerged through troubleshooting and participant suggestions during this pilot that may inform future implementation of VOOF self-collection. Saving and uploading video files was the most common issue. This step requires multiple sequential actions and stable connectivity, likely contributing to the cumbersome usability reported by patients. In future studies, this could be improved by integrating VOOF self-collection into the SUD treatment app or performing it during a video visit (11, 12). Some participants also reported discomfort with how to perform the test and with recording themselves. Prior work has suggested that individualized coaching by study staff and brief “how-to” videos can improve participant comfort with home-based sample collection (15). After this pilot had been underway, Mayo Clinic Labs created an instructional video that participants were able to access. Participants also had access to a study staff member for assistance; however, more proactive communication may have reduced uncertainty. Finally, some participants had difficulty finding private spaces with adequate lighting or a stable surface to position their phone for recording. Study staff attempted to address this on a case-by-case basis by offering suggestions for potential setups. In a broader rollout, another individual could potentially assist with recording; however, this would introduce privacy concerns and may not be desirable for all patients.

Several limitations should be considered. The overall sample was small, uncontrolled, and not powered for comparisons. Recruitment was drawn from a single health system and was convenience-based, preventing quantification of how many people declined to participate. Additionally, the sample was predominantly White with co-occurring mental illness, and may not generalize to a broader SUD population, including those with treatment-refractory symptoms, or to individuals with housing instability or digital literacy issues (40). Implementation outcomes were based on participant-reported measures and did not include objective evaluation by study staff. While a significant portion of our sample received and returned testing kits by mail, recruitment also occurred in a residential unit. Generalizability to the outpatient setting would have been strengthened if all participants had been recruited from home, as location may impact the individual’s testing environment, their level of privacy, internet or cellular service, and kit return logistics. While no statistically significant differences were seen across recruitment sites, this may be secondary to the study being underpowered to detect those differences. Implementation outcomes focused on participant-reported measures and kit return rates. Evaluations by study staff were not assessed, including video assessments, oral fluid sample validity, and testing results. Importantly, participants may have found this acceptable, but executed the test incorrectly, which could prevent downstream use. Qualitative participant feedback was also anecdotal and not obtained in a structured way. Future work, including semi-structured interviews to further explore patient perspectives on recording and self-collection, along with the viewpoints of clinicians, laboratory staff, and biospecimen analysis, would allow for a more comprehensive assessment of implementation barriers and viability.

This early-stage pilot study found that VOOF self-collection was generally perceived as acceptable, appropriate, and feasible among individuals in SUD treatment. Usability scores were below commonly accepted benchmarks; however, several infrastructure and workflow-related factors likely contributed to this, particularly the multi-step video recording and uploading process. As outlined above, integrating VOOF within a broader digital framework, such as a mobile application, may help to mitigate some of these concerns. Further evaluation is required in larger cohorts, potentially with repeated measures, to assess how comfort and usability evolve over time. Additionally, the views of clinicians and study staff, along with an assessment of the end-to-end sample-to-results process, are also needed to fully assess VOOF implementation. As such, conclusions on VOOF self-collection are limited to the patient perspective.

## Data Availability

The raw data supporting the conclusions of this article will be made available by the authors, without undue reservation.
