# Supplementary material for: Implementation outcomes of video-observed oral fluid self-collection for drug testing in substance use disorder treatment: a pilot study
Source: Front Public Health. 2026 May 19;14:1833909. doi: 10.3389/fpubh.2026.1833909 (PMC13228157; doi:10.3389/fpubh.2026.1833909)
Supplement: Supplementary file 1 [file Table_1.DOCX]

Supplementary Table 1: Item-level responses for the System Usability Scale

| Question | Mean (SD) |
| --- | --- |
| I think that I would like to use this system frequently | 2.1 (1.4) |
| I found the system unnecessarily complex | 2.0 (1.3) |
| I thought the system was easy to use | 2.1 (1.3) |
| I think that I would need the support of a technical person to be able to use this system | 2.0 (1.4) |
| I found the various functions in this system were well integrated | 2.4 (1.3) |
| I thought there was too much inconsistency in this system | 1.8 (1.5) |
| I would imagine that most people would learn to use this system very quickly | 2.3 (1.5) |
| I found the system very cumbersome to use | 1.9 (1.5) |
| I felt very confident using the system | 2.3 (1.2) |
| I needed to learn a lot of things before I could get going with this system | 2.0 (1.6) |

Description: Questions were rated on a 5-point Likert scale (Strongly Disagree to Strongly Agree), with higher scores indicating greater perceived usability. Negatively worded items were reverse-coded, consistent with standard scoring, to allow comparison across items.
